# Xanthohumol Protects Against Neuronal Excitotoxicity and Mitochondrial Dysfunction in APP/PS1 Mice: An Omics-Based Study

**DOI:** 10.3390/nu16213754

**Published:** 2024-10-31

**Authors:** Fei-Fei Hu, Shi-Yao Pan, Jin-Yu Chu, Jian-Jun Liu, Ting-Ting Duan, Yu Luo, Wen Zhou, Zhi-Ming Wang, Wei Liu, Yan Zeng

**Affiliations:** 1 Hubei Provincial Clinical Research Center for Alzheimer’s Disease, Wuhan University of Science and Technology, Wuhan 430065, China; hufeifei@wust.edu.cn (F.-F.H.); panshiyao@wust.edu.cn (S.-Y.P.); chujy1316@gmail.com (J.-Y.C.); 17862667717@163.com (T.-T.D.); lylyly@wust.edu.cn (Y.L.); 1532090194@wust.edu.cn (W.Z.); 2418859272@wust.edu.cn (Z.-M.W.); 2 Brain Science and Advanced Technology Institute, Medical School, Wuhan University of Science and Technology, Wuhan 430065, China; 3 Shenzhen Center for Disease Control and Prevention, Shenzhen 518055, China; junii8@126.com

**Keywords:** Alzheimer’s disease, xanthohumol, neuronal protection, mitochondria, multiomics

## Abstract

**Background**: Neuronal excitotoxicity and metabolic decline, which begin in the early stages of Alzheimer’s disease (AD), pose challenges for effective amelioration. Our previous work suggested that the natural compound xanthohumol, the most abundant prenylated flavonoid in hops, prevents memory deficits in APP/PS1 mice; however, the underlying mechanisms remain unclear. **Methods**: This study utilized APP/PS1 mice and cutting-edge omics techniques to investigate the effects of xanthohumol on hippocampal proteome, serum metabolome, and microbiome. **Results**: Our findings revealed that xanthohumol reduces the postsynaptic overexpression of α-amino-3-hydroxy-5-methyl-4-isoxazolepropionic acid, *N*-methyl-D-aspartate, and metabotropic glutamate receptors, but enhances ATP synthesis and mitophagy in the young AD hippocampus. Further mechanistic analyses suggested systemic regulatory effects, particularly on the decreasing glutamate synthesis in the blood and intestines of AD mice following xanthohumol administration. **Conclusions**: These results underscore the potential of xanthohumol in mitigating AD pathology through multifaceted mechanisms, sparking interest and curiosity in its preventive and therapeutic potential in AD.

## 1. Introduction

Alzheimer’s disease (AD) is the most common cause of dementia worldwide, affecting millions of individuals and posing significant challenges to public health [1]. Despite the profound impact of AD, current treatments have not achieved satisfactory therapeutic effects. Recent trials of the Aβ monoclonal antibodies aducanumab [2] and lecanemab [3] have yielded the first clear evidence that reducing soluble and insoluble Aβ in the brains of symptomatic patients can slow disease progression, leading to the first approval for anti-Aβ immunotherapies in the USA under the accelerated approval pathway. However, testing the long-term effects and potential side effects of anti-Aβ immunotherapies in pre-symptomatic populations is the focus of ongoing and future research. Additionally, five other drugs approved by the Food and Drug Administration are used as treatment options for symptomatic relief of AD [4]. However, AD is a complex multifactorial disease. Besides the accumulation of Aβ plaques and tau tangles in the brain [5], factors such as oxidative stress [6], mitochondrial dysfunction [7,8], neuroinflammation [9], and energy metabolism abnormalities [10,11] also contribute to the disease’s progression, even in its early stages. These neurochemical abnormalities in the AD brain warrant renewed emphasis as potential preventive strategies.

The safe and cheap natural dietary compound xanthohumol, a prenylated flavonoid found in abundant amounts in the hop plant *Humulus lupulus* L., stands out as a potential alternative to the aforementioned therapies [12]. It possesses various biological effects, including anti-inflammatory [13] and antioxidant activities [14], as well as promoting mitochondrial biogenesis [15], preventing mitochondrial dysfunction [16], and exhibiting anticancer properties [17]. Our previous study, along with a study by other researchers, demonstrated that xanthohumol improves learning, memory, and cognitive flexibility in both young C57BL/6 and AD mice [18,19]. Moreover, it can inhibit the aggregation of tau protein, decompose fibrils, protect cells from apoptosis induced by tau oligomers [20], reduce tau phosphorylation [21], and prevent Aβ aggregation [22] in the various AD cell models. These findings suggest that xanthohumol is a promising agent for preventing neurodegenerative diseases.

However, existing studies have only explored the neuroprotective effects of xanthohumol against memory deficits and have failed to elucidate the underlying molecular mechanisms in animal models. It is unclear how the systemic regulation of xanthohumol, a multitarget drug, synergizes to promote its neuroprotective effects. Therefore, a comprehensive omics study is crucial for understanding the xanthohumol cross talk between molecular mechanisms and target sites across multiple organs in preventing AD.

To contribute to the development of novel therapeutic strategies for AD that can slow disease progression, we investigated the potential neuroprotective mechanisms of xanthohumol against memory deficits in young APP/PS1 mice using hippocampal proteomics, blood metabolomics, and gut microbiota analyses. Further, we evaluated alterations in the various features associated with AD pathology following xanthohumol treatment.

## 2. Materials and Methods

### 2.1. Animals

Double-transgenic mice (APPswe/PS1de9, APP/PS1) and wild-type (WT) mice were purchased from the Medical Laboratory Animal Center of Guangdong, China. These mice were housed in standard vivarium cages at a Shenzhen Center for Disease Control and Prevention (CDC) specific pathogen-free facility, with ad libitum food and water available on a 12 h dark/light cycle. Two-month-old AD mice and age-matched WT mice on a C57BL6/J background were used in the study.

### 2.2. Xanthohumol Administration and Behavioral Tests

To ensure comparable cognitive function among experimental mice, the novel object recognition (NOR) test [23] was performed to assess short-term memory before the xanthohumol administration. The NOR test was administered by measuring the time that animals spent recognizing and remembering new objects between 8:00 a.m. and 3:00 p.m. for two days: the training and recognition phases. An animal behavior video analysis system (Xeye Aba V3.2, MacroAmbition S&T Development Co., Ltd., Beijing, China) captured and recorded the time each mouse spent on the exploring object. Equal numbers of male and female mice with comparable cognition were randomly allocated to three groups: (a) WT group: WT mice received corn oil (0.1 mL/10 g body weight) (*n* = 11); (b) AD group: AD mice received corn oil (0.1 mL/10 g body weight) (*n* = 10); and (c) AD+Xn group: AD mice received xanthohumol (0.5 mg/kg) (*n* = 9). The corn oil or xanthohumol was administrated by oral gavage each other day for 90 days. The Morris water maze (MWM) test was conducted after completing the 3-month xanthohumol administration to evaluate learning and memory abilities, following the method described in our previously published study [18].

### 2.3. Sample Preparation

Following the behavior test, all mice were anesthetized with an intraperitoneal injection of 10% chloral hydrate (10 mL/kg) in a sterile environment. Hippocampus tissues were extracted from the mice’s brains and immediately frozen at −80 °C. Blood was collected using the eye-picking method. Serum was obtained from the collected blood samples with centrifugation at 3000× *g* for 10 min at 4 °C. The whole intestine was removed under sterile conditions, and fecal samples (1–2 g) of the middle and upper sections of the rectum were placed in an EP tube (1.5 mL) after sterilization and the samples quickly immersed in liquid nitrogen. All samples were stored at −80 °C for the following experiments.

### 2.4. Hippocampal Proteome Analysis

The hippocampus was homogenized in lysis buffer (8 M urea and 1% protease inhibitor cocktail [Roche, Mannheim, Germany]) and lysed for 40 min at 4 °C. Following centrifugation (12,000× *g* for 10 min at 4 °C) and collection of the supernatant, the protein concentration was measured using a bicinchoninic acid protein assay kit in accordance with the manufacturer’s instructions.

Dithiothreitol was added to each sample at a final concentration of 10 mM and incubated at 37 °C for 1 h to open the protein’s disulfide bonds. Iodoacetamide was added at a final concentration of 25 mM and incubated at room temperature for 1 h in the dark to ensure that the protein sample was completely denatured and kept in a reduced state. Urea in the protein extract was diluted to 1 M using phosphate buffer saline, and Trypsin/Lys-C Mix (Promega, WI, USA) was added at a ratio of 1:25 and digested at 37 °C for 12 h. The enzymatic reaction was terminated by heating at 60 °C for 30 min.

The pH value of the sample solution was adjusted to 1–2 with 0.1% trifluoroacetic acid, centrifuged at 12,000 rpm, and the precipitate was discarded. Desalting was performed using an Oasis HLB cartridge (1 mL). Isobaric labeling of peptides was performed using tandem mass tagging (TMT) reagents (Thermo, NJ, USA). The TMT-labeled peptides were subsequently eluted with mobile phase B (0.1% formic acid in acetonitrile) on a high-performance liquid chromatography separation system (UltiMate3000) with gradients from 4% to 6% (within 5 min), 8% to 18% (within 35 min) to 32% within 22 min, up to 95% in 2 min, and maintained at 95% to 5% for the last 4 min, with a flow rate of 0.20 μL/min, and the gradient eluted for 120 min. The resulting peptides were subjected to mass spectrometry (Q-Exactive, Bruker, Bremen, Lower Saxony, Germany) to record the mass-to-charge ratio (*m*/*z*) of each peptide and to obtain mass spectrometry (MS) data. An Xcalibur (v 2.1.2) was used for the MS data acquisition based on data-dependent acquisition mode. The orbital trap was set at a resolution of 60,000 and an *m*/*z* range of 400–1800. Each primary MS scan was followed by 20 secondary MS scans.

Next, the SEQUEST module in Proteome Discoverer 2.1 and the FASTA database (mouse) were used to retrieve and align proteins based on the acquired MS/MS data. Proteins with a false-discovery rate (FDR) confidence of “high” (<1%) were retained for subsequent analysis.

### 2.5. Serum Metabolism Analysis

Metabolites were extracted from 50 μL of serum samples using 50% methanol buffer (200 μL), followed by vortexing for 1 min, incubating for 10 min at room temperature, and then storing at −20 °C overnight (16 h). The next day, the mixture was centrifuged at 13,000× *g* for 20 min at 4 °C. The supernatant was then transferred to a 1.5 mL EP tube and dried in a vacuum centrifuge at 50 °C for approximately 1.5 h. Then, the samples were re-dissolved in 100 μL of sterile water, vortexed for 1 min, and centrifuged at 14,000× *g* for 20 min at 4 °C. The supernatant was then collected for liquid chromatography MS (LC-MS) analysis.

Chromatographic separation was achieved on a Waters ACQUITY UPLC HSS T3 column (100 mm × 2.1 mm, 1.8 µm; maintained at 40 °C; RVC 2–18 CD plus, CHRIST^®^, (Merck Millipore, Burlington, MA, USA)) with a mobile phase delivered at 0.3 mL/min, consisting of (a) ultrapure water and (b) methanol, both containing 0.1% formic acid. The injection volume was 2 μL. The elution gradient program used was as follows: 0–1 min, 5% B; 1–15 min, 95% B; 15–19 min, 99% B; 19–25, 5% B.

The LC-MS data were processed using Compound Discoverer 3.0 software (Thermo Fisher Scientific Inc., Waltham, MA, USA), including baseline correction, peak extraction and alignment, normalization of peak area, comparison, and identification of metabolites. The mass error did not exceed 5.0 ppm, and the minimum peak area was 1,000,000.

### 2.6. Fecal Microbiome Analysis

16S rDNA section sequencing was performed for fecal samples. Briefly, this included sample DNA extraction, PCR amplification and purification, library preparation, database construction, and taxonomic annotation as per our previous study [18].

### 2.7. Differential Analysis

The Wilcoxon rank-sum test was employed for hippocampal protein expression data to detect the differentially expressed proteins (DEPs) between two groups. DEPs were defined as those with adjusted *p* value ≤ 0.05. Based on the protein expression among the WT, AD, and AD+Xn groups, fuzzy c-means clustering was performed using the R 4.3.2 Mfuzz package [24], resulting in eight clusters of proteins with different expression trends.

For gut microbiota data, linear discriminant analysis (LDA) effect size (LEfSe) analysis was performed using LEfSe 1.0 [25] to determine significant differences in microbial community from the phylum to the genus levels. The microbiome diversity analysis assessed the differences in gut microbial communities among various experimental groups. Microbiome diversity was typically defined within (i.e., α-diversity) and between (i.e., β-diversity) groups. The Shannon and Simpson diversity indices are commonly used α-diversity measures to determine how evenly the microbes are distributed in a community. The β-diversity was calculated based on taxonomic metrics (Bray–Curtis dissimilarities calculated from log-transformed operational taxonomic unit [OTU] abundance). Non-metric multidimensional scaling (NMDS) was performed using Bray–Curtis dissimilarities based on the normalized OTU abundance data of each sample [26]. The stress value was calculated, with a value less than 0.2 indicating that the ordination reasonably reflected the actual dissimilarities between samples.

### 2.8. Brain Single-Cell RNA-Seq Data Analysis for AD Mice

Two publicly available 10X genomics single-cell RNA-seq datasets (SRP243446 [5xFAD = 12, WT = 18] and SRP282467 [J20AD = 1, WT = 3]) from mouse hippocampi were analyzed to determine whether each protein cluster was involved in excitatory neurons. Briefly, our previously published SCAD-Brain [27] platform had performed excitatory neuron annotation. Then, to identify excitatory neuron-specific genes, the percentage of excitatory neurons and the remaining neurons with a certain gene expressed and the corresponding average expression were calculated. The expression and percentage were compared to obtain fold change and percentage difference between excitatory neurons and the remaining neurons. A certain gene was considered to have enhanced expression in excitatory neurons when its percentage in excitatory neurons minus that in other neurons was greater than 10%.

### 2.9. Pathway Enrichment and Function Annotation Analyses

For total DEPs detected between two groups, gene set enrichment analysis (GSEA) was performed using the R 4.3.2 clusterProfiler package to identify the biological processes (BP), cellular components (CCs), and molecular functions (MFs) affected by AD pathology or xanthohumol administration.

For each cluster of proteins identified by Mfuzz, the GlueGO plugin in Cytoscape 3.10.0 was utilized to elucidate the BPs enriched in each cluster and their internal relationships. Additionally, to further evaluate whether a sub-process within a big BP was significantly differed between groups, gene set variation analysis (GSVA) score was estimated for each sample using the R 4.3.2 GSVA package [28] based on an associated gene set collected from the Kyoto Encyclopedia of Genes and Genomes (KEGG) database. The Wilcoxon rank-sum test was used to compare differences between groups, with a *p*-value ≤ 0.05 considered statistically significant.

For 1722 metabolites detected in serum, 660 were identified with PubChemID. Based on these PubChemIDs, metabolic pathway enrichment analysis was performed using the online platform MetaboAnalyst 6.0 [29] to determine the categories of metabolites. PICRUSt 1.7.1 [30] was used for gut microbiome data to identify the pathways in the gut affected by the xanthohumol intervention. All the above statistical analyses were performed based on R 4.3.2.

### 2.10. Molecular Docking Analysis Between Xanthohumol/Glutamate and Glutamate Receptors

To assess whether xanthohumol has the potential as a glutamate antagonist to reduce glutamate excitotoxicity by binding glutamate-binding sites at α-amino-3-hydroxy-5-methyl-4-isoxazolepropionic acid receptors (AMPARs), *N*-methyl-D-aspartate receptors (NMDARs), and metabotropic glutamate receptors (mGluRs), the 3D structures of xanthohumol and glutamate were obtained from the PubChem database (https://pubchem.ncbi.nlm.nih.gov/; accessed on 19 July 2024) and were optimized using the Chem3D 21.0.0 software, and energy minimization was performed using Open Babel to ensure proper geometry and minimize potential steric clashes. Crystal structures of glutamate receptors were sourced from the Protein Data Bank (PDB, https://www.rcsb.org/; accessed on 19 July 2024), and are presented in a Supplementary Table S3. The receptors were prepared by removing any co-crystallized ligands, water molecules, and heteroatoms not relevant to the docking study using PyMOL 2.5.5 software. Protonation states and charges were assigned using AutoDockTools Vina 1.5.7 [31]. PyMOL was used to analyze the flexibility of ligand binding to the pocket residues [32]. The top 20 docking models with minimized energy were retained to assess the competitive binding of xanthohumol and glutamate at each glutamate receptor.

### 2.11. Prediction of the Blood–Brain Barrier Permeability of Compounds

Firstly, the simplified molecular input line entry system (SMILES) identifiers of compounds were retrieved from the PubChem database. Then, the SMILES was entered into the Deep-B3 online server [33] (https://cbcb.cdutcm.edu.cn/deepb3; accessed on 19 July 2024) to evaluate the ability of a compound to penetrate the blood–brain barrier (BBB).

## 3. Results

### 3.1. Xanthohumol Represses Hippocampal Neuron Apoptosis in AD Mice

**Figure 1:**
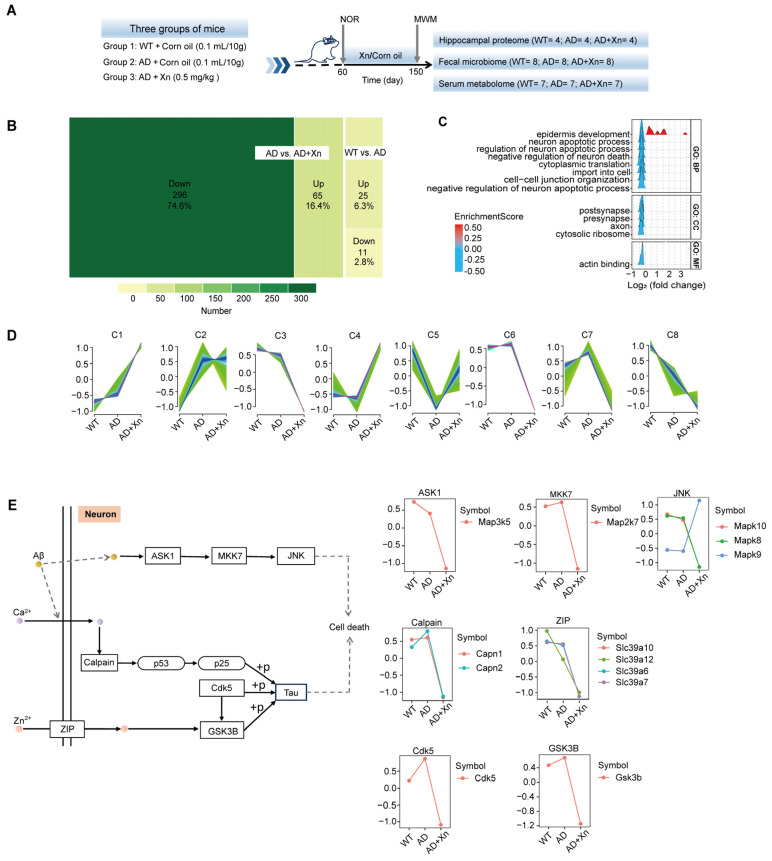
Administration of xanthohumol (Xn) at 0.5 mg/kg/day represses neuron apoptosis pathway in the AD hippocampus. (**A**) Schematic diagram of the experimental design. (**B**) The number and percentage of up- and downregulated proteins in AD vs. WT and AD+Xn vs. AD. (**C**) Gene set enrichment analysis (GSEA) of differentially expressed proteins (DEPs) in AD+Xn compared to AD, including biological processes (BPs), cellular components (CCs), and molecular functions (MFs). (**D**) Trend clustering results of proteins detected in the hippocampi from the WT, AD, and AD+Xn mice using the fuzzy c-means algorithm. Each line represents a gene. The color of the line indicates the membership value, which was generated by mfuzz.plot in the Mfuzz R package. The membership value can vary continuously between zero and one, indicating how well the gene is represented by the cluster. Low values (green) indicate a poor representation of the gene by the cluster, while high values (blue and purple) indicate a strong representation. (**E**) Well-known associations of proteins within the ASK1/JNK and Cdk5/GSK3β cascades with Aβ and tau phosphorylation, as recorded in the KEGG database (details are provided in Figure S1). Expression trends of these proteins across three groups of mice. The Y axis represents the normalized expression value estimated by Mfuzzy.

During the baseline period, mice were randomly assigned to groups according to the novel object recognition test (new object exploration time/total exploration time × 100%) (Figure S1A). Following Xn treatment, spatial learning and memory were assessed using the Morris water maze (Figure S1B–E). Following Xn treatment, spatial learning and memory were assessed using the MWM. Our previous study demonstrated that 3-month xanthohumol treatment of 6-month-old AD mice can significantly improve spatial behavioral deficits at a late age (9 months old) [18]. At an earlier age, this study found that starting xanthohumol at 2 months old can moderately improve the cognitive performance of AD mice at 5 months old (Figure S1A). This is reasonable, as the cognitive deficits in spatial learning and memory of APP/PS1 mice in the MWM began at 7 months [34]. To assess the underlying mechanisms of xanthohumol in preventing cognition deficits and whether xanthohumol exerts its protective effects at a molecular level before significant behavioral impairment occurs, we evaluated the hippocampal proteome, blood metabolome, and intestinal microbiome in the present study (Figure 1A). We identified 36 proteins that were differentially expressed between the hippocampi of AD and WT mice that were fed corn oil. Compared to corn oil, xanthohumol downregulated 296 proteins, but upregulated 65 in the AD hippocampus (Figure 1B). Subsequently, GSEA of xanthohumol-regulated proteins revealed significant negative enrichment of neuronal apoptotic processes (Figure 1C) and subcellular components related to synapses and axons (Figure 1C).

To further elucidate how xanthohumol regulates neuronal apoptosis in the AD hippocampus, we focused on its effects on molecules associated with tau and amyloid pathology (Figure S1). First, we conducted a fuzzy c-means clustering analysis using Mfuzz to identify the proteins responsible for rescuing dying neurons. Among the 4513 proteins detected in the hippocampal samples, we identified six patterns across eight clusters (C1–C8; Figure 1D, Table S1A). Xanthohumol treatment maintained the expression trend from control to AD in C1 and C8, downregulated proteins in C3 and C6, which were moderately altered in AD and significantly upregulated the expression of C4 and C5, which were repressed in the AD hippocampus. Additionally, xanthohumol deactivated the proteins at C7 that were activated in AD mice.

Next, we evaluated the functional roles, biological processes, and their internal relationships reflected by these clustered proteins using KEGG, Gene Ontology (GO) term enrichment analysis, and a network method based on genetic similarity between the biological processes (Table S1B). These results consistently indicated the enrichment of autophagy-associated pathways in C2, C6, and C7 (Table S1B,C), which were downregulated in AD mice treated with xanthohumol.

Finally, we mapped these protein clusters to the molecular pathological pathways involved in AD. We found that xanthohumol inhibited the expression of multiple apoptotic downstream signals of mitogen-activated protein kinases (MAPKs), including apoptosis signal-regulating kinase 1 (ASK1, also known as Map3k5) [35], mitogen-activated protein kinase 7 (MKK7, also known as Map2k7) [36], and c-Jun N-terminal kinase (JNK, including Mapk8/10) [37] (Figure 1E and  Figure S1). Xanthohumol also repressed the expression of calpains (Capn2) and Cdk5, which are related to tau phosphorylation [38,39,40] and upregulated in AD (Figure 1E). These results suggest that xanthohumol administration represses apoptosis and may rescue hippocampal neurons in AD.

### 3.2. Xanthohumol Modulates Mitochondrial Bioenergetics and Mitophagy in the Hippocampi of AD Mice

**Figure 2:**
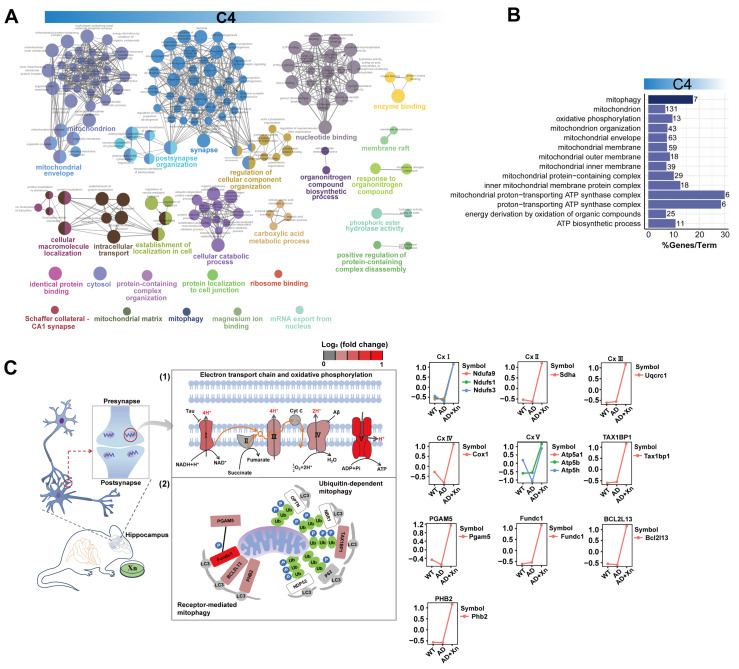
Administration of Xn modulates mitochondrial bioenergetics and mitophagy in AD hippocampus. (**A**) A network showing the internal relationship between GO terms enriched by the C4 proteins. (**B**) The pathways were associated with mitophagy, mitochondrial architecture, and function, the bar chart colors align with those in panel (**A**). The values on the bars represent the number of enriched genes, and the height of the bars indicates the percentage of enriched genes out of the total genes in the pathway. (**C**) Expression trends of proteins that were associated with (1) oxidative phosphorylation and (2) mitophagy. The Y axis represents the normalized expression value estimated by Mfuzzy.

We observed a significant enrichment of C4 proteins involved in mitochondrial structure and energy production processes (Figure 2A,B, Table S1B,C). Concerning mitochondrial structure, xanthohumol modified mitochondrial membrane organization (Figure 2A, Table S1B,C). In terms of mitochondrial function, xanthohumol regulated proton transport, ATP synthesis, and purine nucleotide metabolism (Figure 2A, Table S1B,C). Notably, some C4 proteins were located in the inner mitochondrial membrane and were integral to oxidative phosphorylation complexes (Figure 2C), including complexes I (Ndufa9, Ndufs1, and Ndufs3), II (Sdha), III (Uqcrc), IV (Cox1), and V (Atp5a1, Atp5b, and Atp5h), suggesting that xanthohumol treatment enhanced energy production.

Defective mitophagy in dysfunctional mitochondria is a well-known characteristic of neurodegeneration in AD pathology [41]. We investigated the mitophagy pathway activated in xanthohumol-treated AD hippocampal tissues (Figure 2B) by mapping key factors implicated in mitophagy using the KEGG mitophagy pathway (Figure S2). We discovered that xanthohumol induced receptor-mediated mitophagy instead of ubiquitin-dependent mitophagy by upregulating a series of autophagic adapters (Fundc1, Phb2, and Bcl2l13) that recruit autophagosomes through LC3, a key protein in the autophagy pathway (Figure 2C and  Figure S2). Taken together, xanthohumol may promote ATP synthesis and the mitophagy of dysfunctional mitochondria in the AD hippocampus, suggesting another potential mechanism by which xanthohumol prevents neuronal death by modulating mitochondrial bioenergetics.

### 3.3. Xanthohumol Regulates the Glutamate–Glutamine Cycle in Hippocampal Glutamatergic Synapses

**Figure 3:**
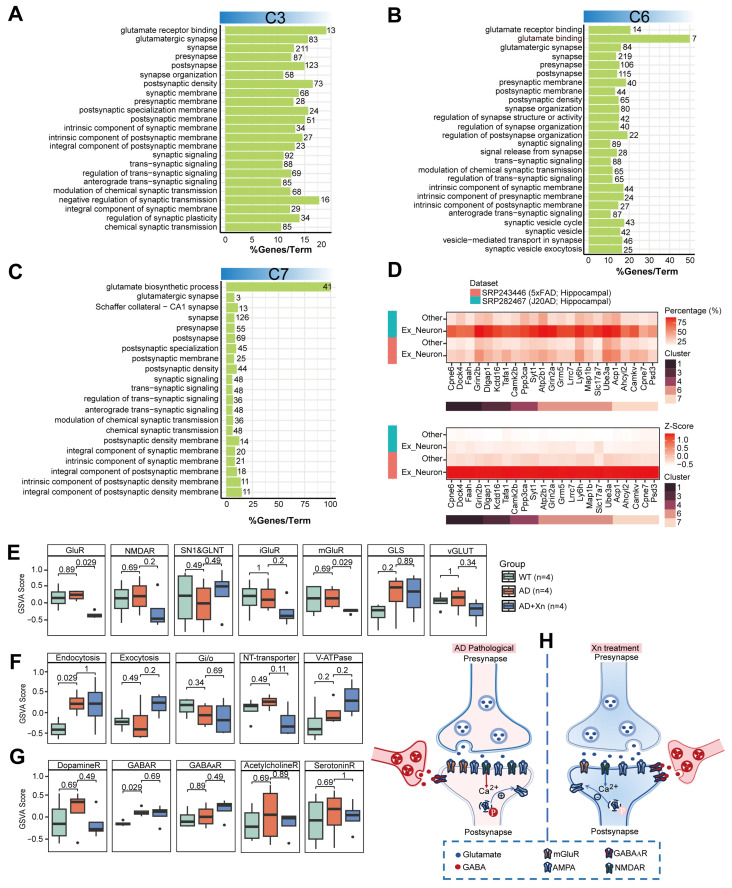
Administration of Xn downregulates C3, C6, and C7 proteins that were involved in the glutamate–glutamine cycle. (**A**–**C**) The number and percentage of protein-coding genes that were associated with glutamatergic synaptic pathways in C3, C6, and C7. The values on the bars represent the number of enriched genes, and the height of the bars indicates the percentage of enriched genes out of the total genes in the pathway. (**D**) The scRNA-seq datasets (SRP243446 and SRP282467) showing the expression levels of 23 genes highly expressed in excitatory neurons across different clusters. GSVA scores of gene sets involved in (**E**) glutamatergic neuron, (**F**) vesicular circulation, and (**G**) other types of neuron, where the points represent outliers. (**H**) Integration of results from panels (**A**–**G**) to provide an overview of receptors on synapses responding to Xn administration.

Besides mitochondrial bioenergetics [42], neuronal excitotoxicity [43] is another mechanism of neuron injury, especially in the early stages of AD pathology. Our functional enrichment analysis indicated that the structure and function of pre- and post-synapses were the major targets through which xanthohumol ameliorated AD pathology. This involved proteins clustered in C3 (Figure 3A), C4 (Figure 2A,B), C5 (Figure S3), C6 (Figure 3B, Table S1), and C7 (Figure 3C, Table S1). Given the diverse protein expression patterns observed across C3–C7, we conducted further investigations to ascertain whether proteins within C3–C7 exert excitatory or inhibitory effects on neuronal activity. Based on two publicly available 10X genomics single-cell RNA-seq datasets (SRP243446 and SRP282467) from the hippocampi of AD mouse models (5xFAD and J20AD), we found 23 genes that were overrepresented in excitatory neurons (Table S2). Among these, 16 were clustered in C3, C6, and C7 (Figure 3D), and their expression was reduced in AD mice treated with xanthohumol. These findings suggest that xanthohumol mitigates AD progression by suppressing excitatory neuronal overactivity during the early stages of AD development. Functional enrichment analyses further corroborated this assertion, revealing that proteins in C3, C6, and C7 were associated with glutamatergic synapse function (Figure 3A–C, Table S1).

To illustrate how these proteins cooperatively decreased the excitability of glutamatergic neurons, we mapped them onto the “glutamatergic synapse” KEGG pathway (Figure S3A). Glutamatergic synapse-specific gene sets were collected for GSVA score estimation (Figure 3E). The results suggested that xanthohumol interrupts the excitability of glutamatergic neurons, mainly by reducing the expression of glutamate receptors on the postsynaptic membrane (Figure S3A). Expression of glutamate receptors, which are overactivated in the AD brain [44], primarily mGluRs (including Grm1/3/5/7/8), was reduced by xanthohumol (Figure 3E). Other ionotropic GluRs (iGluRs), including NMDARs (Grin1/2a) and AMPARs (Gria1/3), as well as factors involved in glutamate packaging into vesicles (vGLUT), were moderately repressed by xanthohumol (Figure S3B), but without statistical significance (Figure 3E). Additionally, nonspecific synaptic vesicle cycle processes, including exocytosis, neurotransmitter transporters, and vacuolar-type adenosine triphosphatase, were moderately regulated by xanthohumol (Figure 3F).

To investigate whether xanthohumol specifically targets glutamatergic neurons instead of other types, the GSVA scores of the receptors on different types of neurons were estimated. The results indicated that the activity of the receptors on cholinergic and serotonergic synapses was not modulated by xanthohumol (Figure 3G). However, xanthohumol slightly repressed the expression of these receptors on dopaminergic neurons (Figure 3G), which is also a neuron-protective event [45]. It moderately increased the expression of gamma-aminobutyric acid (GABA) type A receptor (GABA_A_R) on inhibitory neurons (Figure 3G and  Figure S3C), but not GABA synthesis (Figure 3G and  Figure S3C). These results suggest that xanthohumol leads to the de-excitation of glutamatergic neurons, mainly by inhibiting mGluRs and iGluRs.

Considering the increased permeability of the BBB in AD brains [46], we performed a molecular docking analysis to further investigate whether xanthohumol could directly repress glutamate receptors (Table S3A). The results indicated that xanthohumol has a higher binding affinity for glutamate receptors than glutamate (Table S3B–H). The docking results also explained the inhibition of glutamate receptors (Gria1, Grin2a, Grin1, and Grm7) by xanthohumol through competitive binding with glutamate active sites (Figure S4).

### 3.4. Xanthohumol Affects Gut Microbial Composition and Reduces Gut-Sourced Glutamate Synthesis

**Figure 4:**
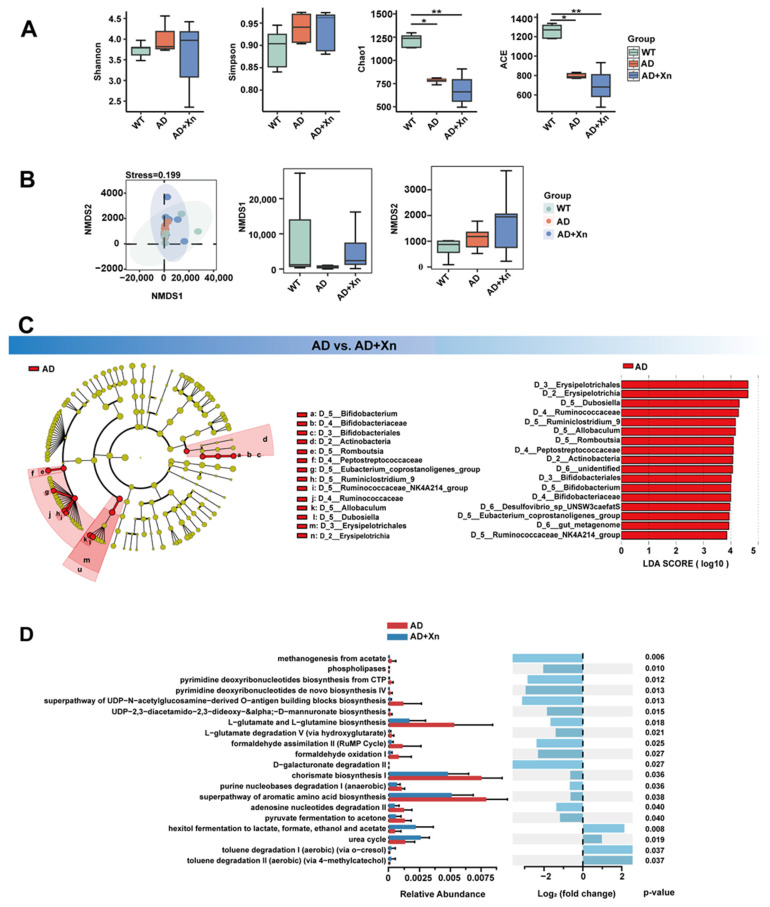
Differential enrichment of gut microbes in AD+Xn and AD groups. (**A**) Comparison of gut microbial community α-diversity, including estimates of community richness (Chao1 and ACE) and community diversity (Shannon, Simpson) across groups (* *p* < 0.05, ** *p* < 0.01, analyzed by *t*-test). (**B**) Comparison of gut microbial β-diversity, including Bray–Curtis principal coordinate analysis (PCoA) and non-metric multidimensional scaling (NMDS) across groups (WT, AD, AD+Xn). (**C**) The gut microbiota was differentially abundant between the AD and AD+Xn mice. The cladogram shows taxonomic levels from phylum to species, with circle size reflecting relative abundance. Red represents groups significantly abundant in AD (linear discriminant analysis (LDA) effect size (LEfSe): *p*-value < 0.05, LDA > 2.0). (**D**) The relative abundance of Kyoto Encyclopedia of Genes and Genomes (KEGG) modules was annotated by PICRUSt2 (v 2.5.3) software. The colors highlight enrichment in the AD (red) and AD+Xn (blue) groups. The differential KEGG pathways were identified with log_2_ (fold changes) and *p*-value < 0.05 between the AD and AD+Xn groups.

Gut microbiota are a potential pathway through which various drugs regulate neuronal function. Therefore, we performed 16S rRNA gene sequencing to examine the effects of xanthohumol on the diversity and composition of the gut microbiota. The Shannon and Simpson diversity indices, which are commonly used α-diversity measures, were not significantly different among the groups (Figure 4A). Additionally, β-diversity increased in AD mice after treatment with xanthohumol (Figure 4B). The community richness indices Chao1 and ACE, which indicate microbial abundance, were lower in AD mice and those treated with xanthohumol (Figure 4A) than in WT mice.

To examine whether the taxa that changed in AD mice treated with xanthohumol played key roles in neuronal protection, we performed LEfSe analysis to identify the differentially over- or under-represented microbial taxa between AD and AD mice treated with xanthohumol (Figure 4C). Consequently, a lower abundance of the phylum Actinobacteria, class Erysipelotrichia, and families Peptostreptococcaceae and Ruminococcaceae was identified in xanthohumol-treated AD mice than in AD mice. Ruminococcaceae-sourced isoamylamine has been known to promote age-related cognitive dysfunction [47]. PICRUSt2 was used to predict changes in metagenomic functions based on the microbiota. The results revealed that glutamate biosynthesis was repressed in xanthohumol-treated AD mice (Figure 4D). However, xanthohumol treatment enhanced the microbiota-mediated urea cycle (Figure 4D).

### 3.5. Xanthohumol Reduces Blood-Derived Glutamate Synthesis

**Figure 5:**
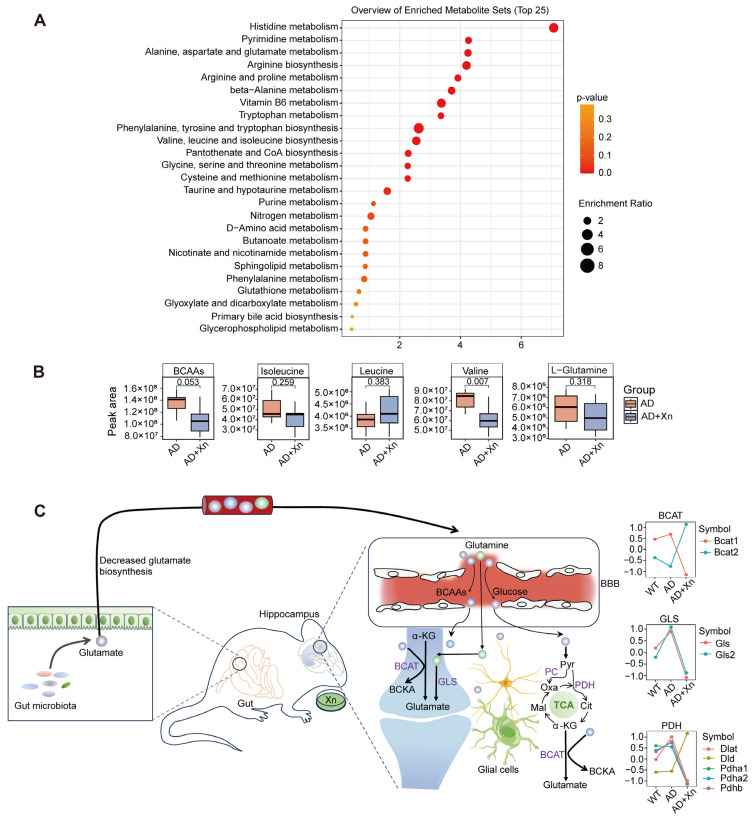
(**A**) The top 25 metabolic pathways enriched by detected metabolites. (**B**) Serum metabolite peak areas of branched-chain amino acids (BCAAs, including leucine, isoleucine, and valine) and L-glutamine were compared between AD and AD+Xn groups. A *p*-value < 0.05 was considered statistically significant. (**C**) Overview of glutamatergic neuron de-excitability by Xn through reducing blood- and gut-derived glutamate synthesis. α-KG, α-ketoglutarate; BCAT, branched-chain aminotransferase; BCKA, branched-chain α-keto acid; GLS, glutaminase; Pyr, pyruvate; Cit, citrate; Mal, malate; Oxa, oxaloacetate; PC, pyruvate carboxylase; PDH, pyruvate dehydrogenase.

As diet has the strongest predictive power in regulating blood metabolites [48], we thus further focused on blood metabolites to investigate evidence supporting the potential of xanthohumol for neuroprotection. Our untargeted metabolomic analysis measured the levels of 660 metabolites, including amino acids, nucleotides, vitamins, and lipids (Table S4). MetaboAnalyst was used to determine the categories of xanthohumol-altered metabolites that were mainly involved in amino acid and nucleotide metabolism (Figure 5A). Notably, xanthohumol dramatically repressed the metabolism of systemic branched-chain amino acids (BCAAs), including valine, leucine, and isoleucine (Figure 5B), which are cognition-related metabolites [49]. They can be transported into the brain and participate in glutamate biosynthesis [50]. Another precursor of glutamate synthesis, blood L-glutamine, which is BBB-permeable (Figure S5), was moderately downregulated by xanthohumol (Figure 5B), although the difference was not statistically significant. Taken together, these results indicate that xanthohumol downregulates blood-sourced glutamate synthesis. Furthermore, it suppresses the expression of hippocampal enzymes associated with glutamate synthesis based on blood-derived metabolites, including branched-chain aminotransferase (BCAT), pyruvate dehydrogenase (PDH), and glutaminase (GLS) (Figure 5C).

## 4. Discussion

The cognitive benefits of xanthohumol in AD mice highlight the need for a deeper understanding of the modifying mechanisms and importance of intervening early in the pathogenic cascade. As described in our multiomics study of young AD mice treated with xanthohumol, we demonstrated a new line of potential neuroprotective mechanisms of xanthohumol in preventing memory deficits, including increased mitochondrial energy metabolism and mitophagy and decreased neuronal excitotoxicity to prevent neuronal death. Our study sheds light on xanthohumol as an alternative therapeutic agent for protecting neurons in AD mice.

The primary goal of AD therapy is to reduce neuronal loss. Neuronal death in AD is a multifaceted process involving a complex interplay of molecular pathways, including Aβ and tau pathology [5], mitochondrial dysfunction [51,52], and neuronal excitotoxicity [53,54,55]. Therapeutic strategies targeting these mechanisms offer promising avenues for disease modification. Our results, along with those of previous studies, confirm the neuroprotective effect of xanthohumol in AD [18,19,20,21,22]. Following preventive treatment with xanthohumol, we observed significant alterations in a long line of proteins associated with neuronal death in the hippocampi of young AD mice. First, the JNK cascade, which is activated by Aβ [56], was repressed. Second, the proteins that lead to tau phosphorylation, including Capn2 [38,39] and Cdk5 [40], were reduced. Although the neuroprotective effects of xanthohumol are well known, this study is the first to elucidate its role in modulating downstream molecular pathways associated with Aβ and tau pathology.

Mitochondrial deficiencies have been identified as the central mechanism in AD brains, leading to mitochondria being explored as a potential therapeutic target [57,58,59]. For instance, Aβ accumulation and tau aggregation trigger defective mitophagy [60,61], while mitophagy stimulation inhibits Aβ and tau pathology and reverses cognitive deficits in AD models [62]. Besides AD, these mitochondrial pathologies are prevalent across a wide range of neurodegenerative diseases [58], suggesting that mitotherapeutics could be valuable for multiple neurodegenerative conditions [63]. We found that a series of adapters (Fundc1, Phb2, and Bcl2l13) involved in receptor-mediated mitophagy [64] were upregulated, indicating the mitophagy pathway in AD hippocampal tissue was induced by xanthohumol treatment. Generally, the activation of mitophagy by xanthohumol is supported by published studies using cell models [65]. Additionally, we found that deficiencies in oxidative phosphorylation in the AD hippocampus could be rescued by xanthohumol through the upregulation of proteins involved in mitochondrial complexes, including complexes I (Ndufa9, Ndufs1, and Ndufs3), II (Sdha), III (Uqcrc), IV (COX1), and V (Atp5a1, Atp5b, and Atp5h). Studies have suggested that these mitochondrial complexes serve as important targets in mitotherapeutics for neurodegenerative diseases, including AD [66] and Parkinson’s disease [67]. Our results suggest that xanthohumol promotes ATP synthesis and mitophagy in dysfunctional mitochondria in the AD hippocampus, suggesting a potential mechanism by which xanthohumol prevents neuronal death through the modulation of mitochondrial bioenergetics.

Neuronal hyperactivity is an early-stage feature consistently shared by patients [68], animals [69], and cell models [70] of AD, progressing to hypoactivity during later stages [71]. Glutamate and its receptors have been implicated in neuronal excitotoxicity [71] and synaptic dysfunction [72,73,74], and represent important targets for therapeutic intervention in AD [73,75,76]. In this study, xanthohumol specifically downregulated the expression of hippocampal glutamate receptors, primarily mGluRs (Grm1/3/5/7/8), followed by iGluRs (NMDARs and AMPAR). Further molecular docking analysis indicated a competitive combination of xanthohumol and glutamate at the active sites of these glutamate receptors. The potential of xanthohumol for AD treatment is notably enhanced when considering the increased permeability of the BBB in AD brains [46], along with evidence suggesting that xanthohumol derivatives are BBB-permeable [22]. From the perspective of systematic regulation, blood-derived glutamine and BCAAs are the main pathways for brain glutamate synthesis, with one-third of the amino groups of brain glutamate derived from BCAAs [77]. Recent studies have reported alterations in blood levels of glutamate [78] and BCAAs [78], which reflect systemic metabolic changes that are strongly correlated with AD [79]. Our analysis of blood metabolites revealed a noteworthy decrease in blood BCAAs and glutamine levels after xanthohumol administration, suggesting that xanthohumol modulates blood-derived glutamate production, which contributes to its neuroprotective effects. Since blood glutamate scavenging has been reported as a potential novel therapeutic approach for other neurodegenerative diseases, such as post-traumatic brain injury anxiety [80] and ischemic stroke [81], our study suggests that xanthohumol may operate through the mechanism of scavenging blood glutamine and BCAAs, positioning it as a potential glutamate-based therapeutic approach for AD.

It is important to acknowledge the limitations of this study. First, the use of animal models may not fully replicate human AD pathology. Second, the bioavailability and pharmacokinetics of xanthohumol require further investigation to optimize its therapeutic efficacy. Third, the effect of xanthohumol in the late stages of AD requires elucidation, considering that neuronal excitotoxicity may not be a problem during this stage. Finally, our findings primarily highlight xanthohumol’s potential neural protective mechanisms without directly linking them to established pathology indicators, which is a valuable focus for our future research. The urgency and importance of addressing these limitations are crucial for advancing xanthohumol as a viable therapeutic option for AD.

## 5. Conclusions

In conclusion, our study provides compelling evidence that xanthohumol exerts neuroprotective effects in young AD mice through the modulation of hippocampal proteomics, blood metabolomics, and gut microbiota. By targeting mitochondrial function and neuronal excitotoxicity, xanthohumol shows promise as a multifaceted therapeutic agent for early AD management and neurodegenerative diseases, warranting further exploration of its multifaceted mechanisms and translational potential.

## Data Availability

The data that support the findings of this study are available from the corresponding author upon reasonable request.

## Supplementary Materials

The following supporting information can be downloaded at https://www.mdpi.com/article/10.3390/nu16213754/s1. Figure S1: Behavioral performance of AD mice and the “Alzheimer’s disease” pathway in the KEGG database; Figure S2: Proteins in C1–C8 were mapped onto the “mitophagy” KEGG pathway using the Pathview R package; Figure S3: Protein expression change in glutamatergic and GABAergic synapses; Figure S4: Molecular docking of xanthohumol and glutamate with glutamate receptors using Autodock; Figure S5: Blood–brain barrier permeability of glutamine predicted by the Deep-B3 online web server; Table S1: Trend clustering and pathway enrichment analysis of hippocampal proteins in WT, AD, and AD+Xn mice; Table S2: Expression and proportions of genes highly expressed in excitatory neurons from AD hippocampus; Table S3: Molecular docking analysis of xanthohumol and glutamate with Gria1, Gria3, Grin1, Grin2a, Grm1, Grm3, and Grm7 receptors; Table S4: Pathway enrichment analysis of serum metabolites.
