# Supplementary material for: Xanthohumol Protects Against Neuronal Excitotoxicity and Mitochondrial Dysfunction in APP/PS1 Mice: An Omics-Based Study
Source: Nutrients. 2024 Oct 31;16(21):3754. doi: 10.3390/nu16213754 (PMC11548031; doi:10.3390/nu16213754)
Supplement: Supplementary file 1 [file nutrients-16-03754-s001.zip › Supplementary_matrials.pdf]

Supplementary Materials

Xanthohumol Protects Against Neuronal Excitotoxicity and Mitochondrial Dysfunction in APP/PS1 Mice: An Omics-Based Study

Fei-Fei Hu[1,2,#], Shi-Yao Pan[1,2,#], Jin-Yu Chu[1,2], Jian-Jun Liu[3], Ting-Ting Duan[1,2], Yu Luo[1,2], Wen Zhou[1,2], Zhi-Ming Wang[1,2], Wei Liu[3*], Yan Zeng[1,2,*]

[1]Hubei Provincial Clinical Research Center for Alzheimer's Disease, Wuhan University of Science and Technology, Wuhan, China

[2]Brain Science and Advanced Technology Institute, Medical School, Wuhan University of Science and Technology

[3]Shenzhen Center for Disease Control and Prevention

#These authors contributed equally to the work and should be regarded as co-first author.

*Corresponding authors: Yan Zeng: zengyan68@wust.edu.cn; Wei Liu: liuwei@wjw.sz.gov.cn.

● Supplementary methods

1. Gene sets for GSVA score calculation

We performed GSVA to estimate the global level of the activity of glutamatergic neuron and other types of neuron. The gene sets used for GSVA score estimation are shown below.

The gene sets used for estimation of GSVA score.

**Description**	**Short name**	**Gene sets (proteins detected in our data were underlined)**
SN1 and GLNT	SN1&GLNT	Slc38a1 and Slc38a2
Glutamate bio-synthesis	GLS	Gls and Gls2
Endocytosis	Endocytosis	Dnm1,Ap2b1,Clta,Dnm2,Dnm3,Ap2a1,Ap2a2,Ap2m1,Ap2s1,Cltc,Cltb
Vacuolar H+-adenosine triphosphatases	V-ATPase	Atp6v1b1,Atp6v0b,Atp6v1a,Atp6v0e,Atp6v0a1,Atp6v0a4,Atp6v0d2,Tcirg1,Atp6v1g3,Atp6v1c2,Atp6v1e2,Atp6v0e2,Atp6v0c,Atp6v0a2,Atp6v1h,Atp6v1b2,Atp6v0d1,Atp6v1e1,Atp6v1f,Atp6v1g2,Atp6v1g1,Atp6v1c1,Atp6v1d
Glutamate packaging into vesicles	vGLUT	Slc17a6, Slc17a7, and Slc17a8
Neurotransmitter transporter	NT-Transporter	Slc18a1,Slc18a3,Slc17a8,Slc6a4,Slc1a2,Slc1a6,Slc1a7,Slc17a6,Slc18a2,Slc32a1,Slc17a7,Slc6a13,Slc6a9,Slc1a1,Slc1a3,Slc6a2,Slc6a1,Slc6a7,Slc6a11
Exocytosis	Exocytosis	Unc13c,Unc13b,Stx2,Stx3,Cplx4,Nsf,Vamp2,Syt1,Rab3a,Rims1,Unc13a,Stxbp1,Stx1a,Stx1b,Snap25,Cplx1,Cplx2,and Cplx3
Guanine nucleotide-binding protein	Gi/o	Gng14,Gnb3,Gng7,Gngt2,Gnai1,Gnai2,Gnai3,Gnao1,Gnb1,Gnb2,Gnb4,Gnb5,Gngt1,Gng10,Gng12,Gng2,Gng3,Gng4,Gng5,Gng13,Gng11
Ionotropic glutamate receptors	iGluR	Gria1,Gria2,Gria3,Gria4,Grin1,Grin2a,Grin2b,Grin2c,Grin2d,Grin3a,Grin3b

Metabotropic glutamate receptors	mGluR	Grm4,Grm6,Grm1,Grm2,Grm3,Grm5,Grm7,and Grm8
Postsynaptic glutamate receptors	Glutamate receptors	Grm4,Grm6,Gria2,,Grin2c,Grin2d,Grin3a,Grin3b,Grm1,Grm2,Grm3,Grm5,Grm7,Grm8,Gria1,Gria3,Gria4,Grin1,Grin2a
Postsynaptic dopaminergic receptors	Dopaminergic receptors	Drd1,Drd5,Cacna1d,Cacna1b,Kcnj5,Kcnj9,Drd3,Drd4,Drd2,Scn1a,Cacna1c,Cacna1a,Kcnj3,Kcnj6
Postsynaptic cholinergic receptors	Cholinergic receptors	Chrm5,Chrm3,Kcnq3,Kcnj12,Kcnj2,Kcnj4,Kcnq1,Kcnq2,Kcnj14,Kcnq5,Kcnq4,Chrm2,Chrnb4,Chrna3,Chrna4,Chrna6,Chrna7,Chrnb2,Cacna1d,Cacna1s,Cacna1f,Chrm1,Kcnj3,Kcnj6,Chrm4,Cacna1c,
Postsynaptic GABAergic receptors	GABAergic receptors	Gabra6,Gabrb2,Gabre,Gabrg1,Gabrp,Gabrq,Gabrr1,Gabrr2,Gabrr3,Cacna1b,Cacna1d,Cacna1s,Cacna1f,Gabbr1,Gabbr2,Gabra5,Gabra1,Gabra2,Gabra3,Gabra4,Gabrb1,Gabrb3,Gabrd,Gabrg2,Gabrg3,Slc12a5,Cacna1a,Cacna1c,Kcnj6
Postsynaptic serotonergic receptors	Serotonergic receptors	Cacna1d,Cacna1s,Cacna1f,Htr2a,Htr2b,Htr2c,Htr3a,Htr3b,Htr4,Htr6,Htr7,Htr1b,Htr1d,Htr1f,Htr5a,Htr5b,Cacna1b,Kcnj3,Kcnj5,Kcnj9,Cacna1c,Htr1a,Cacna1a,Kcnj6
GABA type A receptor	GABA$_A$R	Gabra6,Gabrb2,Gabre,Gabrg1,Gabrp,Gabrq,Gabra5,Gabra1,Gabra2,Gabra3,Gabra4,Gabrb1,Gabrb3,Gabrd,Gabrg2,Gabrg3

● Supplementary figures

Figure S1

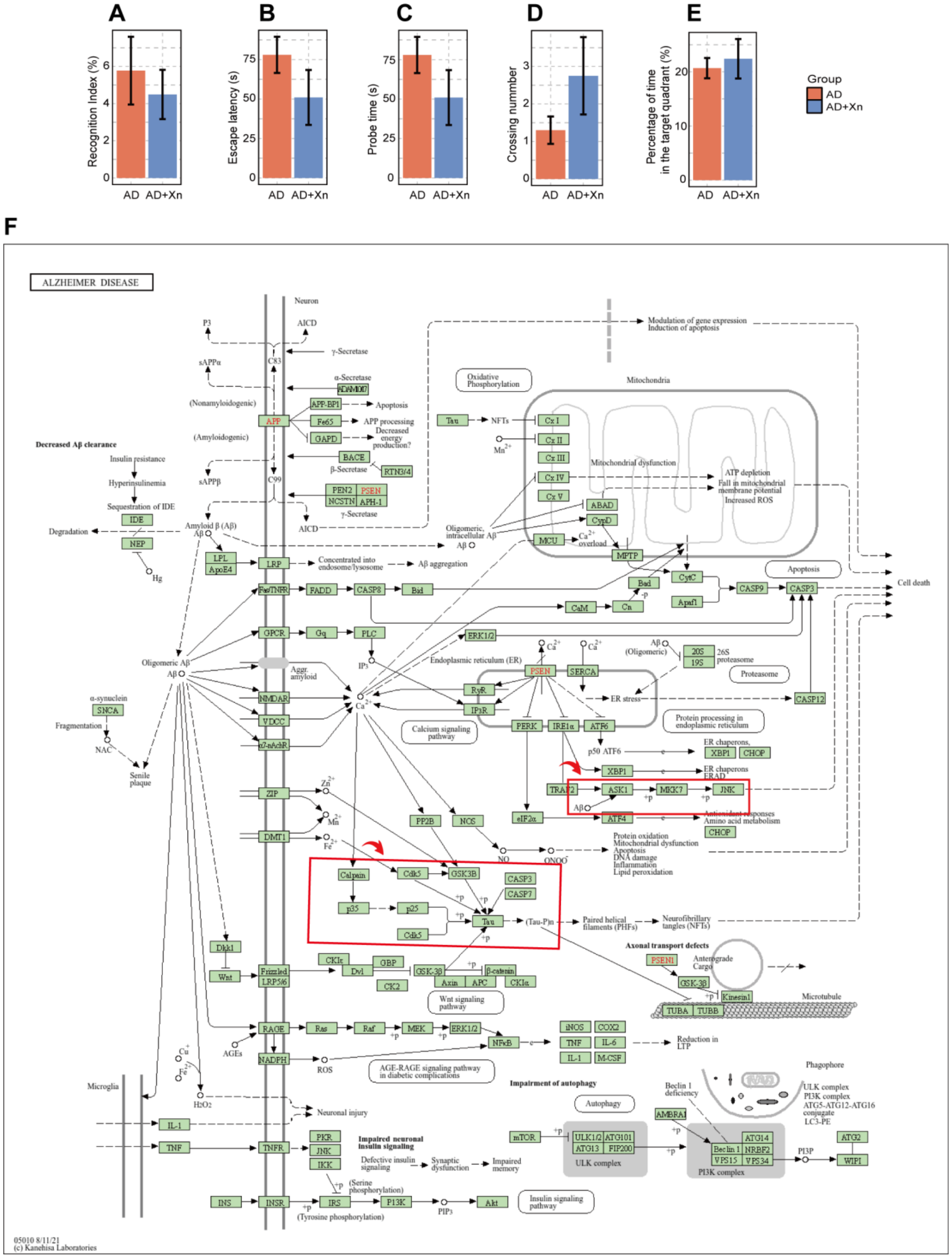

Figure S1. The behavioral performance of AD mice and the 'Alzheimer's disease' pathway in the KEGG database. (A) The baseline novel object recognition test (new object exploration time/total exploration time × 100%) (n = 9-10 per group,

analyzed by t-test, P value > 0.05). **(B-E)** The Morris water maze after Xn treatment (n = 9-10 per group, analyzed by t-test, P value > 0.05). **(F)** The ASK cascade downstream of Aβ and upstream cascade of Tau phosphorylation are highlighted in red.

Figure S2

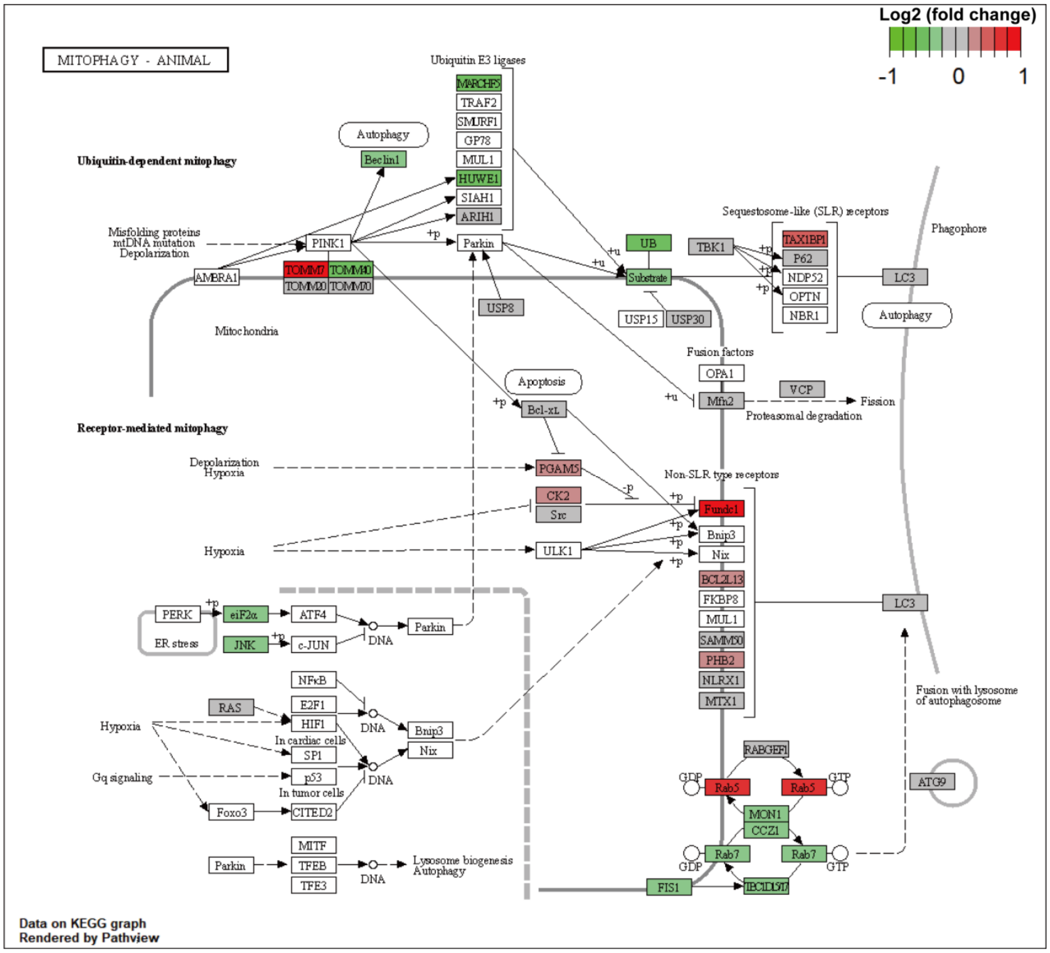

Figure S2. Proteins in C1-C8 were mapped onto the 'mitophagy' KEGG pathway using the Pathview R package. The colors red and green indicate up- and down-regulation of proteins in AD+Xn mice compared to AD.

Figure S3

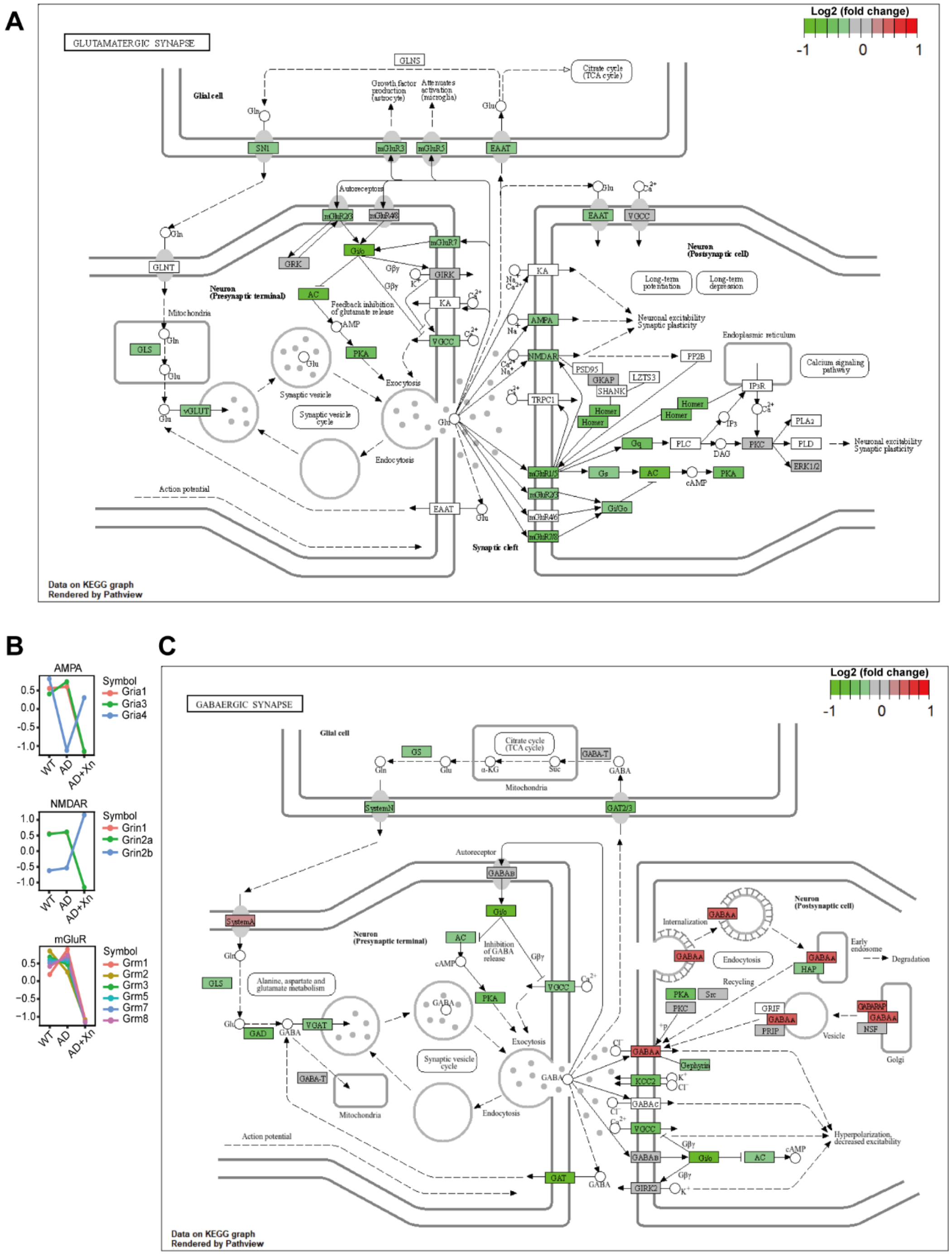

Figure S3. The protein expression change in glutamatergic and gabaergic synapses. (A) The C3/6/7 proteins were mapped onto the 'glutamatergic synapse' KEGG pathway. **(B)** Expression patterns of proteins involved in AMPA, NMDA, and mGluR across WT, AD, and AD+Xn mice. The Y axis are the normalized expression value estimated by Mfuzzy. **(C)** Proteins in C1-C8 were mapped onto the 'gabaergic synapse' KEGG pathway.

Figure S4

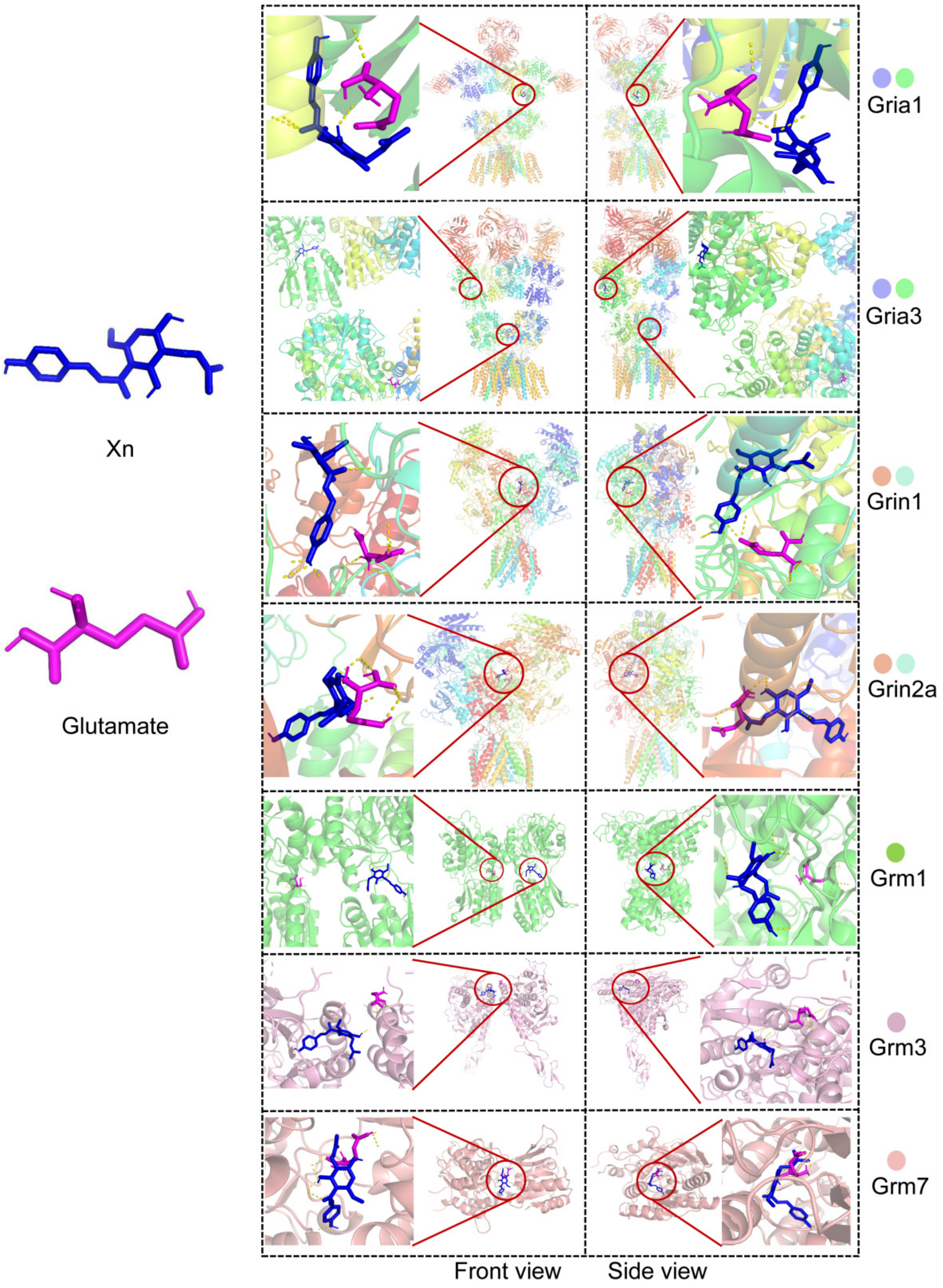

Figure S4. Molecular docking of xanthohumol (Xn) and glutamate with glutamate receptors using Autodock. The glutamate receptors include AMPAR (Gria1 and Gria3), NMDAR (Grin1 and Grin2a), and mGluR (Grm1, Grm3, and Grm7). The three-dimensional structure of Xn (purple) and glutamate (blue) are shown in the stick representation. Hydrogen bonds indicate as dashed lines.

Figure S5

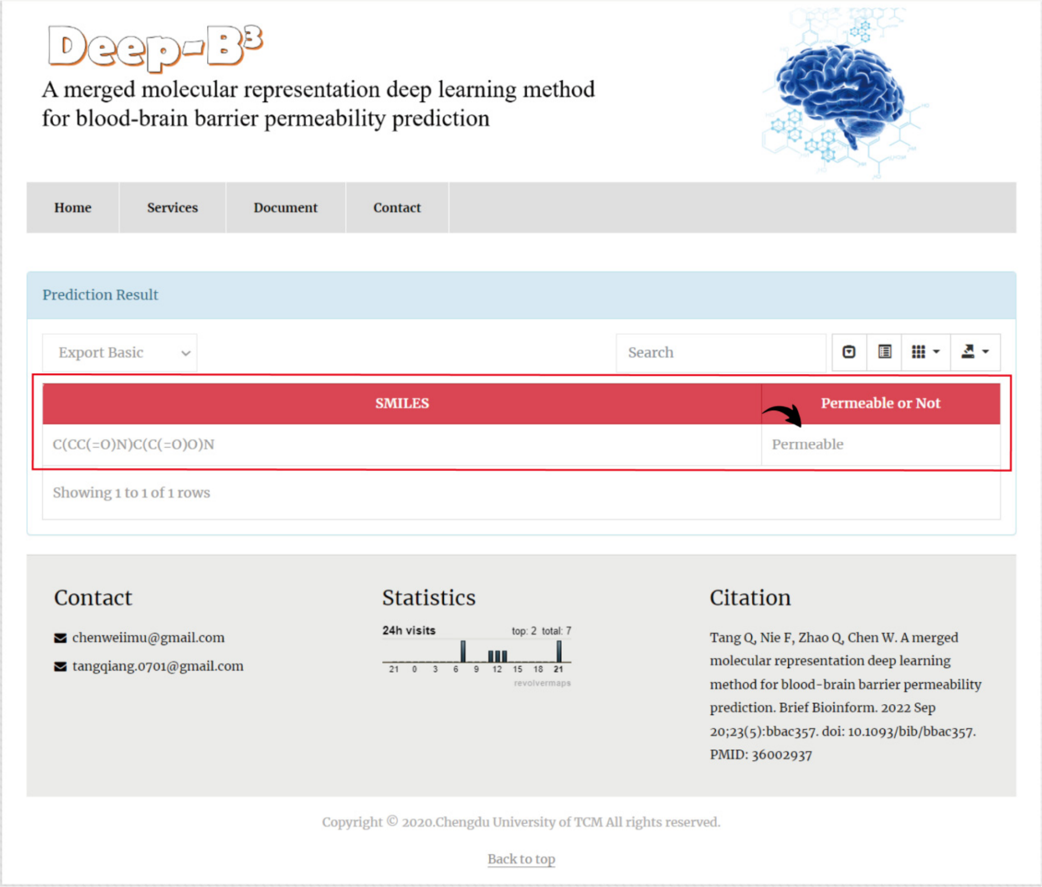

Figure S5. The blood-brain barrier permeability of glutamine predicted by Deep-B[3] online web server.
