# Supplementary figures and images for: Xanthohumol Protects Against Neuronal Excitotoxicity and Mitochondrial Dysfunction in APP/PS1 Mice: An Omics-Based Study
Source: Nutrients. 2024 Oct 31;16(21):3754. doi: 10.3390/nu16213754 (PMC11548031; doi:10.3390/nu16213754)

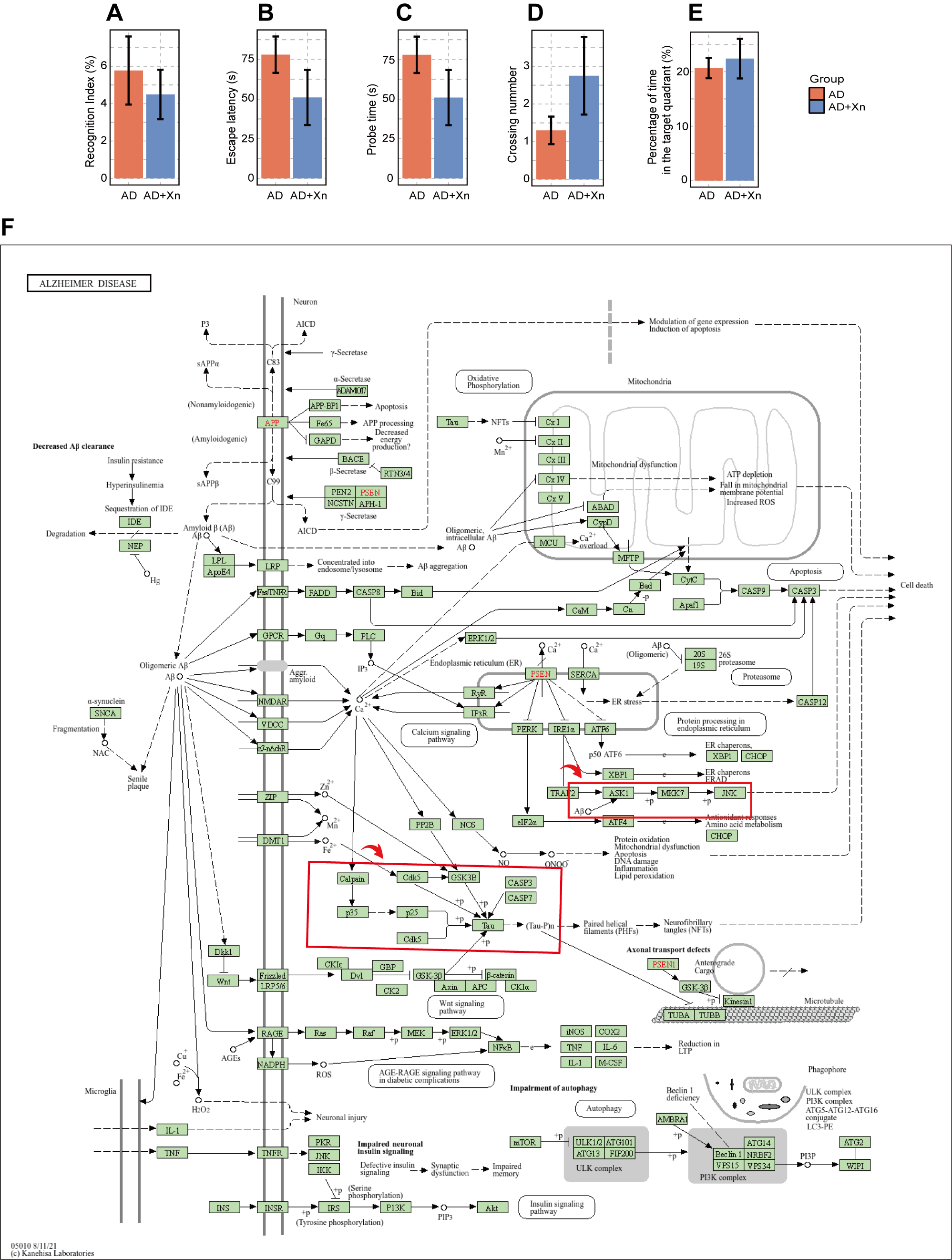

Supplement: Supplementary file 1 [file nutrients-16-03754-s001.zip › FigureS1.tif]

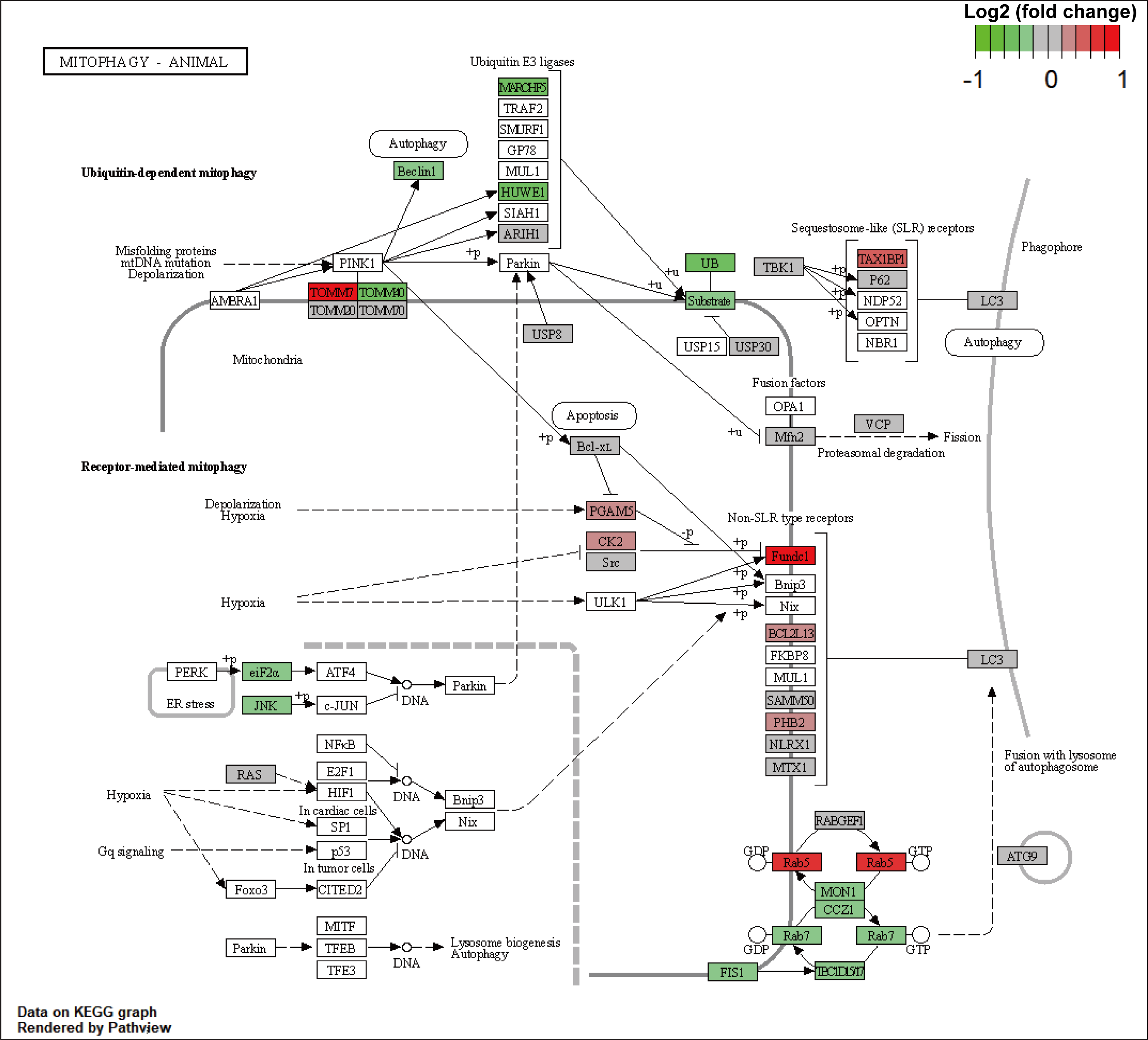

Supplement: Supplementary file 1 [file nutrients-16-03754-s001.zip › FigureS2.tif]

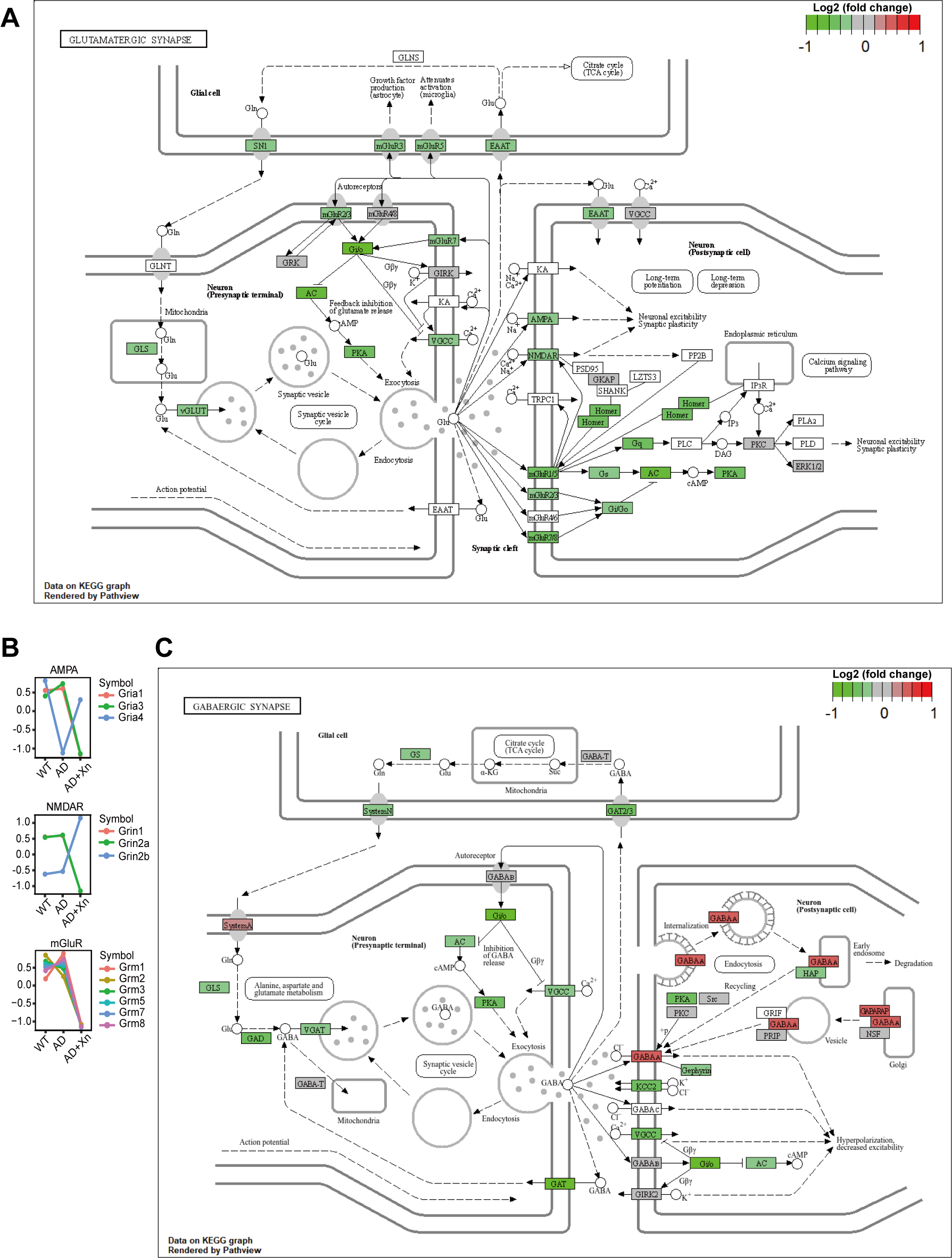

Supplement: Supplementary file 1 [file nutrients-16-03754-s001.zip › FigureS3.tif]

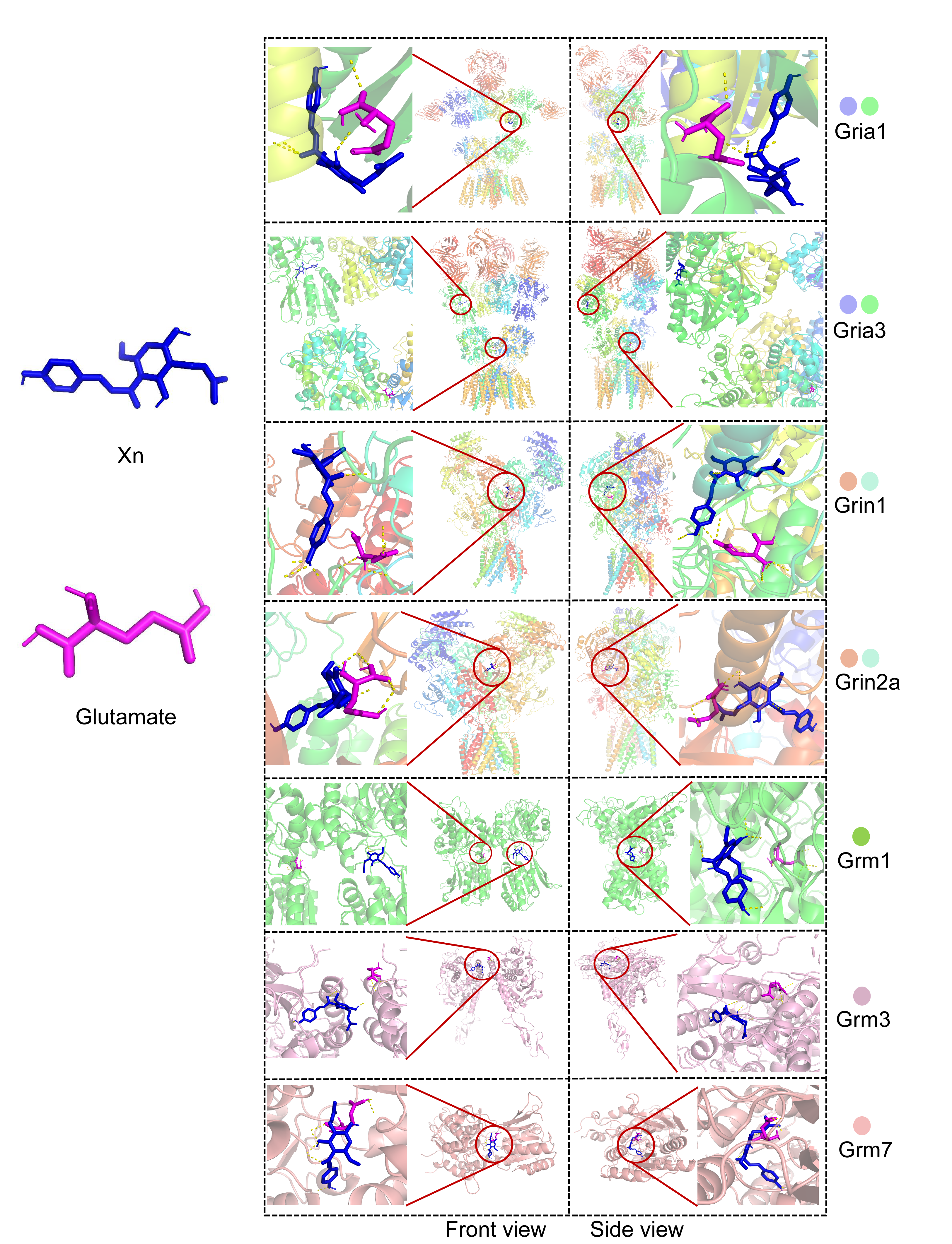

Supplement: Supplementary file 1 [file nutrients-16-03754-s001.zip › FigureS4.tif]

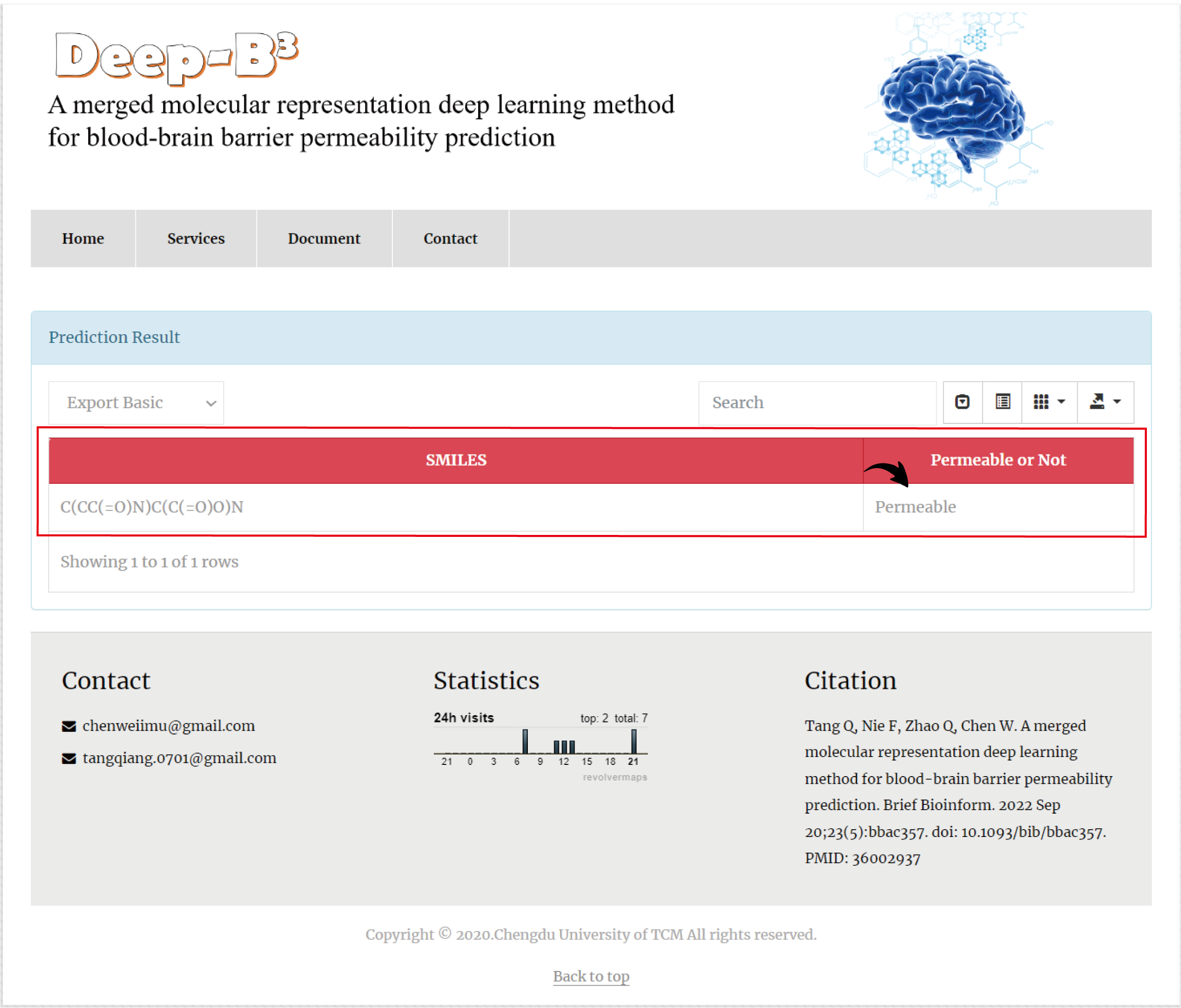

Supplement: Supplementary file 1 [file nutrients-16-03754-s001.zip › FigureS5.tif]
